# Evaluating Wideband Tympanometry Absorbance Changes in Cochlear Implant Recipients: Mechanical Insights and Influencing Parameters

**DOI:** 10.3390/jcm13175128

**Published:** 2024-08-29

**Authors:** Rahel Bertschinger, Christian von Mitzlaff, Marlies Geys, Ahmet Kunut, Ivo Dobrev, Dorothe Veraguth, Christof Röösli, Alexander Huber, Adrian Dalbert

**Affiliations:** Department of Otorhinolaryngology, Head & Neck Surgery, University Hospital Zurich, University of Zurich, 8091 Zurich, Switzerland

**Keywords:** cochlear implant, wideband tympanometry, absorbance, mechanical changes

## Abstract

**Background**: Cochlear implant (CI) electrode insertion can change the mechanical state of the ear whereby wideband tympanometry absorbance (WBTA) may serve as a sensitive tool to monitor these mechanical changes of the peripheral auditory pathway after CI surgery. In WBTA, the amount of acoustic energy reflected by the tympanic membrane is assessed over a wide frequency range from 226 Hz to 8000 Hz. The objective of this study was to monitor changes in WBTA in CI recipients before and after surgery. **Methods**: Following otoscopy, WBTA measurements were conducted twice in both ears of 38 standard CI recipients before and in the range of 4 to 15 weeks after CI implantation. Changes from pre- to postoperative absorbance patterns were compared for the implanted as well as the contralateral control ear for six different frequencies (500 Hz, 750 Hz, 1000 Hz, 2000 Hz, 3000 Hz, 4000 Hz). Furthermore, the influence of the time point of the measurement, surgical access, electrode type, sex and side of the implantation were assessed for the implanted and the control ear in a linear mixed model. **Results**: A significant decrease in WBTA could be observed in the implanted ear when compared with the contralateral control ear for 750 Hz (*p* < 0.01) and 1000 Hz (*p* < 0.05). The typical two-peak pattern of WBTA measurements was seen in both ears preoperatively but changed to a one-peak pattern in the newly implanted ear. The linear mixed model showed that not only the cochlear implantation in general but also the insertion through the round window compared to the cochleostomy leads to a decreased absorbance at 750 and 1000 Hz. **Conclusions**: With WBTA, we were able to detect mechanical changes of the acoustical pathway after CI surgery. The implantation of a CI led to decreased absorbance in the lower frequencies and the two-peak pattern was shifted to a one-peak pattern. The result of the linear mixed model indicates that WBTA can detect mechanical changes due to cochlear implantation not only in the middle ear but also in the inner ear.

## 1. Introduction

A cochlear implant (CI) can restore hearing in patients with severe to profound hearing loss. In recent years, the criteria for cochlear implantation have been broadened to include patients with residual low-frequency hearing [1,2]. However, postoperative reduction or loss of residual hearing occurs in a substantial number of patients [3]. Therefore, it is crucial to understand the underlying mechanisms and mechanical changes leading to such hearing loss in order to improve surgical techniques and patient outcomes.

Previously published results suggest that CI electrode insertion can have effects on stapes displacement, potentially affecting the preservation of residual low-frequency hearing [4]. However, other studies found no such effect and showed that the inner ear pressure and stapes velocity to acoustic stimulation remained largely unchanged post-implantation, indicating minimal impact on middle and inner ear mechanics [5,6]. Independent from cochlear implantation, reinforcing the round window (RW) membrane significantly increased intracochlear sound pressure at frequencies below 2 kHz [7]. This effect was further evaluated with changes in the differential pressure (Pdiff) across the basilar membrane, which correlates closely with hearing sensation: RW reinforcement led to a decrease in Pdiff of 11 dB at 700–800 Hz [8]. A previous clinical capsule report with five participants linked cochlear implantation to a decreased wideband tympanometry absorbance (WBTA) of low frequencies [9]. This aligns with the finding that cochlear implantation increased average wideband power reflectance [10]. Another study found decreased mid- and high-frequency acoustic absorbance in unilaterally implanted children [11].

WBTA is a useful tool to assess the peripheral auditory pathway because, unlike classic tympanometry at 226 Hz, it provides energy absorbance spectra over the range of 226 Hz to 8000 Hz, offering additional information about the mechanical state of the middle and inner ear. A typical WBTA response curve shows a two-peak pattern arising from the natural resonance frequencies of the middle ear (around 0.8–1.2 kHz) and the ear canal (around 2–3 kHz). Different ear pathologies alter the natural resonance frequency of the peripheral auditory pathway. Stiffness-dominated pathologies, such as otosclerosis, increase the natural frequency and decrease the transmission of lower frequencies [12,13,14,15]. In contrast, mass-dominated pathologies, such as middle ear effusion decrease the natural resonance frequency, leading to reduced transmission of higher frequencies and consequently decreased high-frequency WBTA [16]. Furthermore, WBTA spectra are altered also in some inner ear conditions such as superior semicircular canal dehiscence [15,17] or inner ear malformation [18].

Although there are indications that a CI leads to mechanical changes in the inner and/or middle ear, which could influence residual hearing, the contributing factors and their specific impacts remain unclear. The present study aimed to evaluate WBTA changes in a larger cohort of CI recipients and investigate the influence of age, sex, time point of the postoperative measurement (days after surgery), side of implantation, electrode type, and route of insertion. This comprehensive analysis seeks to provide better insight into the mechanical changes induced by cochlear implantation and their correlation with residual hearing.

## 2. Materials and Methods

### 2.1. Study Design

This prospective study was approved by the Ethical Committee of Zurich (KEK-ZH-Nr. 2021-00437) and conducted in concordance with the declaration of Helsinki.

Bilateral WBTA measurements were performed during pre- and postoperative clinical routine visits. The postoperative WBTA was measured four to 15 weeks after cochlear implantation. Otoscopy was performed prior to all WBTA measurements to exclude ear canal obstruction as well as to assess middle ear and tympanic membrane conditions. Additionally, demographic and audiological data, as well as information about the CI electrode type and route of insertion was collected for further analysis.

### 2.2. Participants

A total of 45 adult CI recipients with no history of otologic middle ear conditions in the ear that was to be implanted consecutively provided consent. Seven enrolled participants were excluded from the analysis (dropout (n = 2), no labels for “side of measured ear” (n = 1), measurement artifacts (n = 1), missing values (n = 2), otosclerosis (n = 1)). Seven participants had a CI on the contralateral ear. These data were only used for the linear mixed model but were not included as the control in the statistical analysis. Two participants (n = 2) had a perforation of the tympanic membrane on the contralateral ear and were not included as control ears. In the end, a total of 38 implanted and 29 control ears were included in the analysis.

### 2.3. Measurement Protocol

WBTA measurements were conducted using the Interacoustics Titan tympanometer (Version 1.10.14, Interacoustics A/S, Middelfart, Denmark) and the corresponding Titan Suite software (Version 3.4.1, Interacoustics A/S, Middelfart, Denmark). The energy absorbance of 107 frequencies between 226 Hz and 8 kHz was measured using a click tone as a stimulus. The absorbance was calculated by inverting the energy reflectance, measured by the tympanometer. Bilateral pre- and postoperative measurements were performed at least twice. In the case of unclear or noisy measurements, e.g., in the case of ear canal leakage, measurements were repeated until two reproducible results were obtained. For each measurement, absorbance was displayed at the peak pressure, which is the tympanometric pressure at which absorbance peaked between 376 and 2000 Hz.

### 2.4. Cochlear Implantation

Cochlear implantation was performed via the facial recess approach using the devices “SlimJ” or “MidScala” from Advanced Bionics (Advanced Bionics LLC, Valencia, CA, USA), “CI612” or “CI622” from Cochlear (Cochlear Limited, Sydney, Australia), or “Flex 28 S-Vector” from MEDEL (MEDEL, Innsbruck, Austria). The electrode array was inserted either through the round window (RW) or via cochleostomy of the basal turn; in one case, a different insertion method (extended RW access) was used. Full insertion was achieved in all patients except one, and no complications were reported.

### 2.5. Audiological Assessment

Air- and bone conduction were assessed in accordance with ISO 8253-1:2010 [19].

The formula for hearing preservation (HP) proposed by the HEARRING group was adapted and used to quantify the frequency-specific loss of residual hearing [20].

Proposed formula for qualitative HP classification by the HEARRING group:

(1) $$HP=\left(1-\left(\frac{\left(PTApost-PTApre\right)}{\left(PTAmax-PTApre\right)}\right)\right)\times 100$$

where PTApost is the postoperatively measured pure tone average, PTApre is the pure tone average measured preoperatively, and the PTAmax is the limit of the used audiometer.

To quantify the hearing loss at a specific frequency the formula was adapted to classify the frequency-specific relative hearing loss (RHL):

(2) $$RHL=\left(\frac{\left(PTLpost-PTLpre\right)}{\left(PTLmax-PTLpre\right)}\right)\times 100$$

where PTLpost is the postoperatively measured pure tone level of a specific frequency, PTLpre is the pure tone level of a specific frequency measured preoperatively, and the PTLmax is the limit of the used audiometer for the specific frequency.

### 2.6. Data Analysis

A total of 38 participants were included in the analysis. To extract the WBTA data, the two measurements were averaged and stored for further analysis with MATLAB R2022b (MathWorks, Inc., Natick, MA, USA). Statistical analysis and visualization were performed using R (Version 4.3.1, RStudio 2023.09.1). The used frequencies for statistical analysis are 0.5, 0.75, 1, 2, 3, and 4 kHz, as these frequencies are around the typical WBTA peaks and are tested in routine clinical audiological assessments. To compare pre- and postoperative data and the implanted and unimplanted ear, a paired two-tailed Student’s *t*-test (R t.test{stats}, Version 4.3.1) was performed and plotted using ggplot{ggplot2} (R, Version 3.4.4). To identify peak absorbance and their corresponding frequencies, the function “find_peaks{ggpmisc}” (R, Version 0.5.5) was used on the averaged data. The analysis of the peak pressure to ensure the absence of middle ear pathologies was conducted by performing an ANOVA (R, aov{stats}, Version 4.3.1) between the four conditions (OP vs. Control and pre- vs. postoperative). To assess the correlation between WBTA measurements and hearing threshold pre- and postoperatively, a linear model was used with the R package lm{stats} (R, Version 4.3.1). A linear mixed model to investigate possible confounding factors such as time point of the measurement, route of insertion, electrode type, sex and age of the participants, as well as side of the implantation, was performed with lmer{lme4} and lmerTest{lme4} (R, Version 1.1-35.3).

## 3. Results

### 3.1. Study Population

Characteristics of all the participants included in the analysis are summarized in Table 1. Seven participants who were previously implanted with a CI on the contralateral ear are summarized separately compared to the non-contralateral CI group. The mean age of the total included participants was 63.1 years (SD = 11.8), and 57.9% (n = 22) were female and 42.1% (n = 16) were male. Of the participants, 24 (63.3%) were implanted on the right side and 14 (36.8%) on the left side. A straight electrode was implanted in 22 (57.9%) cases, the other 16 (42.1%) participants received a precurved CI electrode. The route of insertion was through the RW in the majority (68.4%, n = 26) and, for 12 (31.6%) participants, with a cochleostomy.

None of the included participants showed any middle ear pathologies or relevant postoperative changes, as assessed by otoscopy and analysis of the measured peak pressures. The mean of the peak pressures was around ambient pressure for all conditions (OP preoperative: Mean = 0.39 daPa, SD = 27.0; Control preoperative: Mean = 7.42 daPa, SD = 30.93; OP postoperative: Mean = −1.99 daPa, SD = 60.16; Control postoperative: Mean = 6.50 daPa, SD = 37.52). No difference was found between the peak pressures in the different groups (OP vs. Control) at the two time points (pre- vs. postoperative) (ANOVA: *F* = 0.445, *p* = 0.72, Supplementary Materials Figure S1).

### 3.2. No Group Differences in Baseline WBTA

**Figure 1:**
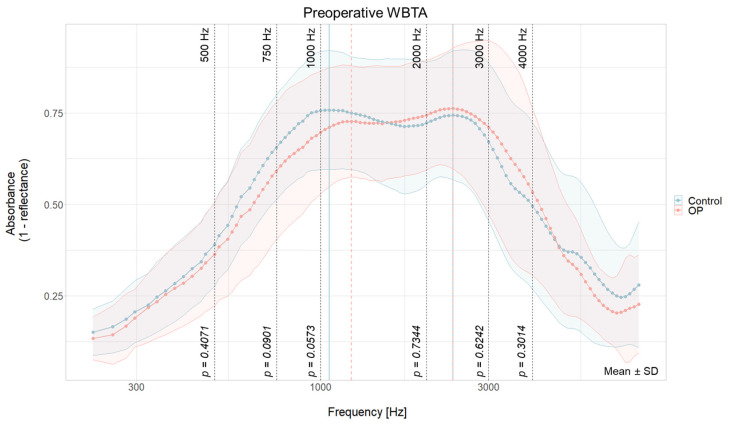
Comparison of preoperative WBTA absorbance spectra of the OP ear (light red) and non-implanted ears (Control, light blue). Peaks are indicated by vertical lines in light red (OP ear) and light blue (control ear). Frequencies for statistical analysis are shown with black dashed lines and their corresponding *p*-value. Data are shown as means for each measured frequency ± SD. Mean and SD with results of the paired two-tailed test are summarized in Supplementary Materials Table S1.

Preoperative WBTA for the implanted ear (n = 38, OP) and for the non-CI control ear (n = 29, Control) are displayed in Figure 1. The y-axis of the graph shows the energy absorbance ranging from 0 (no absorbance) to 1 (complete absorbance). The spectra are the grand mean for each measured frequency with standard deviation. In both groups, the absorbance pattern shows two peaks. The first peak is at 1059 Hz for the contralateral ear and at 1224 Hz for the to-be-implanted ear (Table 2). These peaks are five measured frequencies apart and are both around the typical middle ear resonance frequency (0.8–1.2 kHz). A second peak was identified at 2378 Hz for both the ipsi- and the contralateral ear and lays around the natural resonance frequency of the ear canal (2–3 kHz). Lower absorbance can be seen at the low and high end of the frequency range.

No statistical difference between preoperative WBTA measurements of control and OP ear could be found at the statistically tested frequencies (500 Hz: *t*(27) = −0.84, *p* = 0.41; 750 Hz: *t*(27) = −1.76, *p* = 0.09; 1 kHz: *t*(27) = −1.98, *p* = 0.06; 2 kHz: *t*(27) = 0.34, *p* = 0.73; 3 kHz: *t*(27) = 0.495, *p* = 0.62; 4 kHz: *t*(27) = 1.05, *p* = 0.30).

### 3.3. Decreased Absorbance in Lower Frequencies in CI Ear

**Figure 2:**
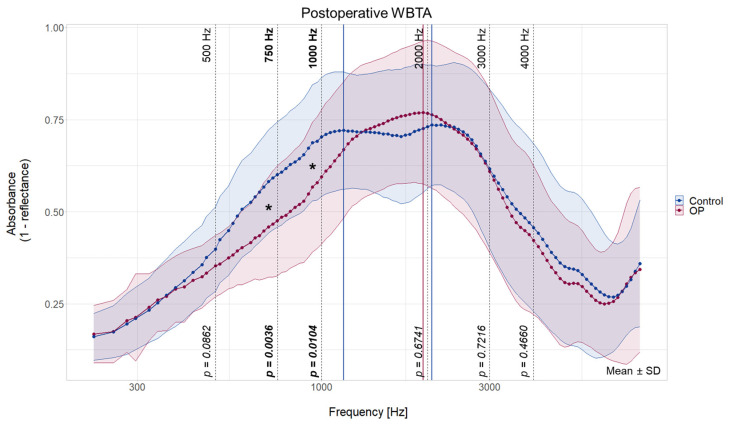
Graphical presentation of postoperative WBTA absorbance spectra of the implanted ears (OP, in red) and non-implanted ears (Control, in blue). Peaks are indicated by vertical lines in dark red (OP ear) and dark blue (control ear). Frequencies for statistical analysis are shown with black dashed lines and their corresponding *p*-value. An asterisk indicates significant frequencies (*). Data are shown as means for each measured frequency ± SD. Mean and SD with results of the paired two-tailed test are summarized in Supplementary Materials Table S1.

The postoperative WBTA results show a decreased absorbance on the newly implanted ear for 750 Hz (*t*(27) = 3.18, *p* < 0.01) and 1000 Hz (*t*(27) = 2.74, *p* < 0.05) when compared with the contralateral non-implanted ear (Figure 2). No difference in WBTA could be found in all other statistically tested frequencies (500 Hz: *t*(27) = 1.78, *p* = 0.09; 2 kHz: *t*(27) = −0.42, *p* = 0.67; 3 kHz: *t*(27) = 0.35, *p* = 0.72; 4 kHz: *t*(27) = 0.74, *p* = 0.47).

The typical two-peak pattern could be found for the postoperative WBTA measurements for the non-implanted ear (peak 1: 1155 Hz, peak 2: 2058 Hz, Table 2). In the CI ear, the absorbance pattern shifted from two peaks preoperatively to a single peak at 1943 Hz postoperatively (Table 2).

### 3.4. In-Group Comparison of the WBTA Pre- vs. Postoperatively

**Figure 3:**
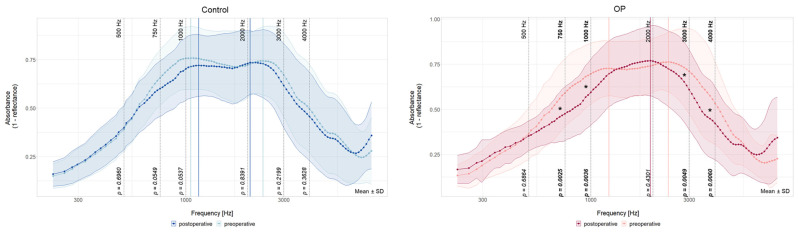
Comparison of pre- and postoperative WBTA absorbance spectra of the non-implanted ears (Control, **left**) and implanted ears (OP, **right**). Preoperative measurements are shown in a lighter color, postoperative WBTAs are displayed in a darker color in both graphs. Peaks are indicated by vertical lines in red (OP ear) and blue (control ear). Frequencies for statistical analysis are shown with black dashed lines and their corresponding *p*-value. An asterisk indicates significant frequencies (*). Data are shown as means for each measured frequency ± SD. Mean and SD with results of the paired two-tailed test are summarized in Supplementary Materials Table S1.

To show the difference between the two measured time points within a group, WBTA absorbance spectra before and after cochlear implantation are displayed in Figure 3 with the control ear (n = 29) on the left and the implanted ear (n = 38) on the right side.

Both pre- and postoperative WBTA in the control ear had a two-peak pattern. The first peak changed slightly from 1059 Hz preoperatively to 1155 Hz (+96 Hz) postoperatively, which is a change of 3 measured frequencies. The second peak shifted minus 5 measured frequencies from 2378 Hz to 2058 (−320 Hz) (Table 2). Although there are some visual differences, no significant changes could be detected in any of the statistically tested frequencies (500 Hz: *t*(27) = −0.39, *p* = 0.70; 750 Hz: *t*(27) = 2.17, *p* = 0.06, 1 kHz: *t*(27) = 2.32, *p* = 0.05, 2 kHz: *t*(27) = −0.21, *p* = 0.84; 3 kHz: *t*(27) = 1.25, *p* = 0.22; 4 kHz: *t*(27) = 0.93, *p* = 0.36).

The right graph in Figure 3 compares the absorbance on the implanted side before and after the CI implantation. Postoperatively, the WBTA is decreased at 750 Hz (*t*(37) = 3.24, *p* < 0.01), 1 kHz (*t*(37) = 3.10, *p* < 0.01), 3 kHz (*t*(37) = 2.99, *p* < 0.01), and 4 kHz (*t*(37) = 2.92, *p* < 0.01) but not at the other two statistically tested frequencies (500 Hz: *t*(38) = 0.41, *p* = 0.69; 2 kHz: *t*(37) = −0.80, *p* = 0.43). Additionally, the preoperative two-peak pattern changed to a one-peak pattern postoperatively. The first peak of the preoperative measurements is not present in the WBTA of the newly implanted ear. The preoperative second peak at 2378 Hz shifted postoperatively minus 7 measured frequencies (−435 Hz) to 1943 Hz (Table 2).

### 3.5. Relationship between WBTA and Acoustic Hearing

**Figure 4:**
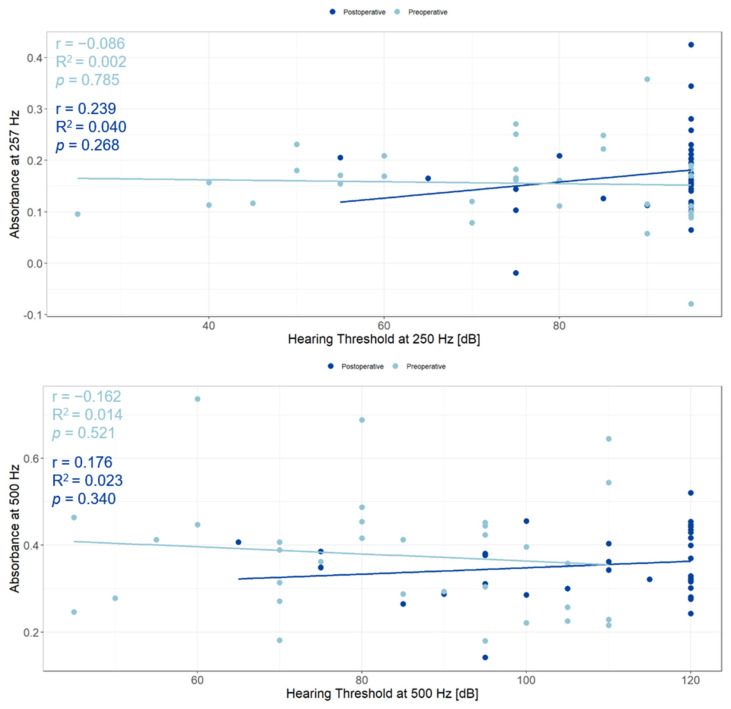
Graphical representation of the correlation between absorbance and the hearing thresholds at 250 Hz (**top**) and 500 Hz (**bottom**). Preoperative data are presented in light blue, postoperative measurements are displayed in dark blue. Not heard stimuli at the maximum output of the audiometer (250 Hz: 90 dB, 500 Hz: 115 dB) are represented as 95 dB for 250 Hz and 120 dB for 500 Hz. The fitted lines of the linear mixed models and their descriptive values are displayed in light blue for preoperative measurements and dark blue for postoperative data.

To assess the correlation between absorbance and hearing level, a linear model was used for pre- and postoperative data (Figure 4). Since residual hearing was often only present at low frequencies in the participants in this study, the hearing threshold at 250 Hz with absorbance at 257 Hz and hearing levels at 500 Hz with absorbance at 500 Hz were investigated. The 257 Hz absorbance measurement was used as it was the closest available frequency to 250 Hz. There were no correlations between the absorbance and the hearing thresholds at 250 Hz (preoperative: r = 0.086, R^2^ = 0.002, *p* = 0.785; postoperative: r = 0.239, R^2^ = 0.040, *p* = 0.27) or 500 Hz (preoperative: r = 0.162, R^2^ = 0.014, *p* = 0.52; postoperative: r = 0.176, R^2^ = 0.023, *p* = 0.34).

**Figure 5:**
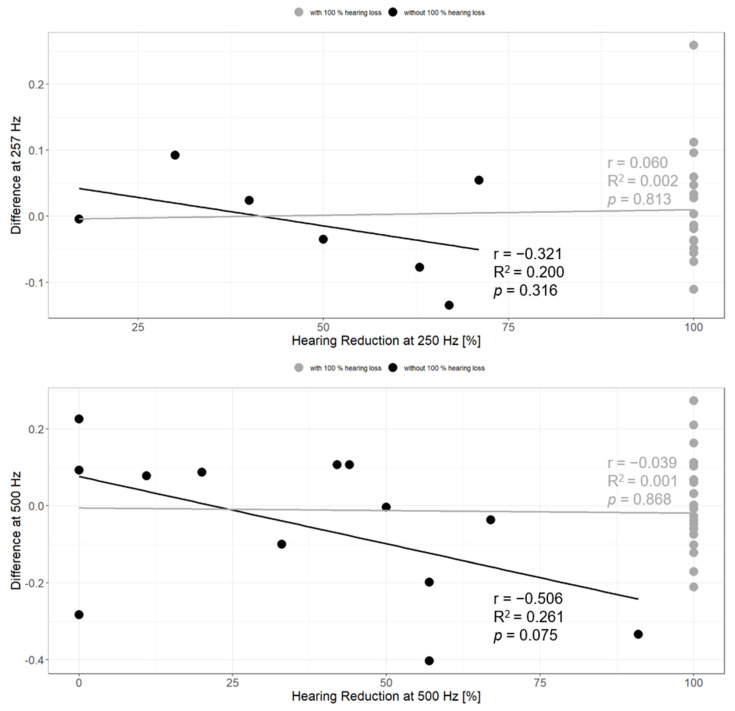
Plot of the change in absorbance and the relative hearing loss at 250 Hz (**top**) and 500 Hz (**bottom**). The change in absorbance is presented as difference between pre- and postoperative WBTA whereas 0 indicates: preoperative absorbance = postoperative absorbance, a positive value: preoperative absorbance < postoperative absorbance, or a negative value: preoperative absorbance > postoperative absorbance. The gray lines are the fitted lines of the linear models when the values of 100% relative hearing loss (indicated as gray dots) are included. In black are the fitted lines of the linear models only with values of a relative hearing loss smaller than 100% (presented as black dots).

Further, we investigated the correlation between the change in absorbance and the relative hearing loss from pre- to postoperative measurements (Figure 5). When including all relative hearing losses from 0% (no loss of residual hearing) to 100% (complete loss of residual hearing), no correlation can be found at 250 Hz (r = 0.060, R^2^ = 0.002, *p* = 0.81) or at 500 Hz (r = 0.039, R^2^ = 0.001, *p* = 0.87). If we exclude all participants with a complete loss of residual hearing (100%) from this analysis to avoid a ceiling effect, we can find a trend of a negative correlation between the change in absorbance and the relative hearing loss at 250 Hz (r = −0.321, R^2^ = 0.200, *p* = 0.32). With a correlation coefficient of r = −0.506, the relationship between the difference in absorbance and the relative hearing loss is even stronger at 500 Hz (R^2^ = 0.261, *p* = 0.08) but not significant.

### 3.6. Contributing Factors

To investigate the postoperative absorbance changes in the implanted ears further, the time point and group (“Condition”: Control preoperative, Control postoperative, OP preoperative, OP postoperative), route of insertion (RW, anterior cochleostomy), electrode type (straight, precurved), sex, side of the implantation, and time point of second measurement (days after surgery) has been assessed and compared in a linear mixed model for 500 Hz, 750 Hz, 1 kHz, 2 kHz, 3 kHz, and 4 kHz.

Only for 750 Hz and 1000 Hz could a significant influence of parameters on WBT absorbance be found (Table 3). The condition “OP postoperative” significantly decreases the WBTA by −0.12 for 750 Hz (*p* < 0.001) and by −0.11 for 1000 Hz (*p* < 0.01). Additionally, the RW insertion decreases the WBTA significantly by −0.17 at 1000 Hz (*p* < 0.05). At 750 Hz, there is a trend that the insertion through the RW also decreases WBTA (estimate = −0.04, *p* = 0.095). No other parameter in the linear mixed model had a significant effect at 750 or 1000 Hz, and none of the parameters had an influence on WBTA in any of the other tested frequencies (0.5, 2, 3, 4 kHz).

## 4. Discussion

### 4.1. Changes in WBTA after Cochlear Implantation

The present study aimed to evaluate WBTA changes in CI recipients, correlate these with hearing change, and further investigate the influence of various parameters, such as electrode type and route of insertion. Consistent with the findings of previous studies, we observed decreased acoustic absorbance in lower frequencies after cochlear implantation. This resembles otosclerotic ears [12,13,14,15] and suggests that the presence of the electrode in the cochlea leads to increased stiffness, although the underlying mechanism is not fully understood.

A possible explanation for the observed WBTA changes could be mechanical alterations within the inner ear. To investigate the influence of inner ear changes on middle ear function and establish potential links between cochlear implantation and increased stiffness, several laser Doppler vibrometry studies were previously conducted. These studies, focusing on RW and stapes movement, produced inconsistent findings. Intraoperative measurements showed that RW and stapes mobility were not altered immediately after insertion of the CI electrode, indicating that the mechanical behavior of the cochlear fluids does not change post-implantation [6]. Similarly, no relevant mechanical changes in terms of intracochlear pressure and stapes velocity were found after cochlear implantation [5]. Conversely, other teams reported variable changes in stapes displacement before and after cochlear implantation [4].

While previous studies have shown mixed results regarding the connection between inner ear changes and middle ear function, our findings suggest a potential association between WBTA changes and the function of the RW membrane. This association is bolstered by our multivariable analysis, which indicates a significant correlation between electrode insertion through the RW and decreased WBTA at 1 kHz. In the literature, both immediate and long-term changes in RW impedance are discussed. Immediate alterations include round window sealing after electrode insertion or occlusion of the cochlear duct by the electrode presence. Long-term effects on RW impedance include intracochlear fibrosis and secondary bone growth. A recent study on deceased cochlear implant recipients found complete RW coverage by fibro-osseous tissue in 85% of cases, with the shortest implantation duration being 12 months [21]. In our study, the latest WBTA measurement was taken at 3.5 months post-implantation, showing stable changes during this period (27 to 105 days after surgery (Median = 37 days, Mean = 45.3 days) analyzed with a linear mixed model. In contrast, another study observed changes over a six-month period, with a trend toward further reduction in the acoustic absorbance [22]. Further studies with extended follow-up durations are warranted to better understand these changes. Another possible mechanism could be the reinforcement of the RW membrane, which has been linked to increased intracochlear sound pressure. This could potentially contribute to the observed mechanical changes and decreased acoustic absorbance in lower frequencies [7].

Other potential factors contributing to increased stiffness include the presence of the electrode lead and increased volume of the middle ear cavity following the induction of the facial recess and mastoid cavity [9,10,23]. However, these explanations focused solely on the middle ear and do not account for the impact of RW insertion on WBTA, which is significant at 1000 Hz. A trend is also observed at 750 Hz, although it does not reach statistical significance. Further investigations with a larger study population are merited.

Similar to other teams, we observed a great variability of absorbance at high frequencies [9,11]. In our within-subject analysis, these absorbance changes at high frequencies reached statistical significance. However, no differences were found in the comparison of postoperative measurements. Furthermore, in the linear mixed model analysis, significant correlation was found only in low frequencies, suggesting that the absorbance changes at high frequencies reflect natural variability rather than an effect of cochlear implantation. One possible explanation for this broad spectrum of high-frequency absorbance variability is substantial variability in middle ear cavity volume [24].

### 4.2. Influence of WBTA Changes on Residual Hearing

In the previous section, the focus was on WBTA changes and inner ear mechanics, particularly in correlation with changes in the RW. We could not identify any other parameter that correlated significantly with WBTA in the linear mixed model. To gain further insights into the effect of changes in WBTA, we correlated absorbance with hearing thresholds. We found no correlation between the hearing thresholds at 250 and 500 Hz and their corresponding absolute absorbance, neither preoperatively nor postoperatively. A previous study found a correlation between acoustic stimulation and changes in differential pressure (Pdiff) across the basilar membrane, which is closely related to hearing sensation. RW reinforcement resulted in an average decrease in Pdiff of up to 11 dB at 700–800 Hz [8]. Therefore, we correlated the difference in WBTA (preoperative–postoperative) with the loss of residual hearing. A substantial number of patients lost all residual hearing postoperatively and were excluded from the analysis due to the ceiling effect. Although the correlation is not significant, there is a trend indicating that a negative difference in absorbance (decreased postoperative WBTA) is correlated with a higher loss of postoperative residual hearing. These results suggest that the increased stiffness caused by CI implantation led to reduced air conduction but needs to be further investigated.

### 4.3. Limitations

One limitation of this study is the uneven distribution between RW insertion and cochleostomy, which may affect the validity and generalizability of our findings. Having a larger and evenly distributed number of insertion routes would validate the significant changes observed.

Another limitation is the variability in the timing of postoperative measurements across patients, which could contribute to inconsistencies in our findings. Although measurements were taken at least 4 weeks post-implantation—allowing for the resolution of immediate postoperative changes such as middle ear effusion—standardizing follow-up intervals would improve the accuracy of long-term effect assessments. Further studies with consistent and extended follow-up periods could provide clearer insights into the sustained impact of cochlear implantation on wideband tympanometry (WBTA) and residual hearing.

To address these limitations, future research should aim for a larger, more balanced sample and standardized and prolonged follow-up intervals. Additionally, investigating the effects of endoscopic-assisted or robotic-assisted cochlear implantation compared to traditional techniques could offer valuable insights into how different surgical methods affect WBTA and hearing outcomes.

## 5. Conclusions

The present study aimed to evaluate WBTA changes in CI recipients, and we found that cochlear implantation leads to increased stiffness on the implanted side. Our findings indicate a potential link between WBTA changes and RW membrane function, suggesting that mechanical alterations in the inner ear, such as RW reinforcement and/or intracochlear fibrosis, could contribute to increased stiffness and reduced acoustic absorbance.

Although no significant correlation was found between hearing thresholds at 250 and 500 Hz and their corresponding absorbance, either pre- or postoperatively, a trend indicated that decreased postoperative WBTA correlates with higher residual hearing loss. This suggests that increased stiffness from cochlear implantation may reduce air conduction.

Overall, our study supplements previous research by providing new insights into the mechanical and acoustic effects of cochlear implantation. It suggests that cochlear implantation could lead to mechanical changes in the inner ear that alter middle ear function, measurable by WBTA. Additionally, we found indications that these changes can also influence air conduction.

## Figures and Tables

**Table 1:** Description of the characteristics of all in the analysis included participants (N = 38) and the subgroups with (N = 7) or without (N = 31) a contralateral CI. No statistically significant differences were found between the sub-groups. Variable distributions are reported as n (%) unless otherwise specified. Abbreviation: RW = round window, SD = standard deviation.

	Total (N = 38)	No Contralateral CI (N = 31)	With Contralateral CI (N = 7)
**Age**			
Mean (SD)	63.1 (11.8)	63.6 (12.2)	61.0 (10.8)
Median [Min, Max]	61.0 [34.0, 82.0]	64.0 [34.0, 80.0]	55.0 [53.0, 82.0]
**Sex**			
Female	22 (57.9%)	17 (54.8%)	5 (71.4%)
Male	16 (42.1%)	14 (45.2%)	2 (28.6 %)
**Postop. Time point (days after OP)**			
Mean (SD)	45.6 (18.0)	45.7 (19.6)	45.0 (7.68)
Median [Min, Max]	37.0 [27.0, 105]	36.0 [27.0, 105]	48.0 [34.0, 54.0]
**Side of implantation**			
Left	14 (36.8%)	9 (29.0%)	5 (71.4%)
Rigth	24 (63.2%)	22 (71.0%)	2 (28.6 %)
**Electrode type**			
Precurved	16 (42.1%)	14 (45.2%)	2 (28.6 %)
Straight	22 (57.9%)	17 (54.8%)	5 (71.4%)
**Insertion**			
Cochleostomy	12 (31.6%)	10 (32.3%)	2 (28.6 %)
RW	26 (68.4%)	21 (67.7%)	5 (71.4%)

**Table 2:** Overview of WBTA peaks: Overview of the WBTA peaks for the control ear (left) and the OP ear (middle) and the difference between them with their corresponding frequency, indices (derived from the numbering of measured frequencies), and the pre- to postoperative change for each parameter for peak 1 (top) and peak 2 (bottom).

		Control		OP		Difference (Control vs. OP)	
		Frequency	Index	Frequency	Index	Frequency	Index
Peak 1	Preoperative	1059 Hz	37	1224 Hz	42	**+165 Hz**	**+5**
	Postoperative	1155 Hz	40	-	-	**-**	**-**
	Change	+96 Hz	+3	-	-		
Peak 2	Preoperative	2378 Hz	65	2378 Hz	65	**0**	**0**
	Postoperative	2058 Hz	60	1943 Hz	58	**−115 Hz**	**−2**
	Change	−320 Hz	−5	−435 Hz	+7		

**Table 3:** Table with the results from the linear mixed model for the tested frequencies (0.5, 0.75,1, 2, 3, 4 kHz). The model was made with the dependent variable absorbance, the fixed effects (condition, sex, side, electrode type, insertion, age, days after surgery), and the random effect of the participant (ID). The intercept and only the parameters that are significant in any of the tested frequencies are presented. Abbreviations: RW = round window, ns = not significant, *** *p* < 0.001, ** *p* < 0.01, * *p* < 0.05, ∙ *p* < 0.1.

Linear Mixed Model Fixed Effects: Condition, Sex, Side, Electrodetype, Insertion, Age, Days after Surgery Random Effect: Participant							
Frequency	Parameter	Estimate	Std. Error	df	t Value	Pr(<|t|)	
500 Hz	Intercept	0.2759	0.07664	33.70	3.601	0.00101	**
	OP postoperative	−0.04377	0.02434	103.0	−1.798	0.07508	∙
	RW Insertion	−0.02683	0.04557	31.50	−0.583	0.56016	ns
750 Hz	Intercept	0.4479	0.1199	32.87	3.737	0.000708	***
	OP postoperative	−0.119974	0.033677	99.131489	−3.562	0.000567	***
	RW Insertion	−0.126833	0.073565	30.028891	−1.724	0.094973	∙
1000 Hz	Intercept	0.506528	0.13221	32.844087	3.831	0.000545	***
	OP postoperative	−0.105709	0.033218	98.365972	−3.182	0.001956	**
	RW Insertion	−0.170700	0.082006	30.346332	−2.082	0.045915	*
2000 Hz	Intercept	0.5653909	0.1494511	32.2508813	3.783	0.000636	***
	OP postoperative	0.0277305-	0.0367911	99.5998430	0.754	0.45279	ns
	RW Insertion	−0.0731738	0.0897201	30.6410767	−0.816	0.42104	ns
3000 Hz	Intercept	0.4990750	0.1971817	32.2091739	2.531	0.0165	*
	OP postoperative	−0.0177946	0.0419342	98.5756814	−0.424	0.67224	ns
	RW Insertion	0.0187486	0.1166916	30.7764810	0.161	0.87340	ns
4000 Hz	Intercept	0.3789150	0.1912172	33.0597188	1.982	0.0559	∙
	OP postoperative	−0.0325083	0.0391552	98.9464383	−0.830	0.4084	ns
	RW Insertion	−0.0789805	0.1147044	31.6396881	−0.689	0.4961	ns

## Data Availability

Authors agree to make data and materials supporting the presented results available upon reasonable request.

## Supplementary Materials

The following supporting information can be downloaded at: https://www.mdpi.com/article/10.3390/jcm13175128/s1, Figure S1: Graphical representation of pre- and postoperative peak pressures; Table S1: Mean and SD with results of the paired two-tailed *t*-test for all statistically tested frequencies.
