# Supplementary material for: Evaluating Wideband Tympanometry Absorbance Changes in Cochlear Implant Recipients: Mechanical Insights and Influencing Parameters
Source: J Clin Med. 2024 Aug 29;13(17):5128. doi: 10.3390/jcm13175128 (PMC11395939; doi:10.3390/jcm13175128)
Supplement: Supplementary file 1 [file jcm-13-05128-s001.zip › jcm-3139891-supplementary.pdf]

Supplementary Materials

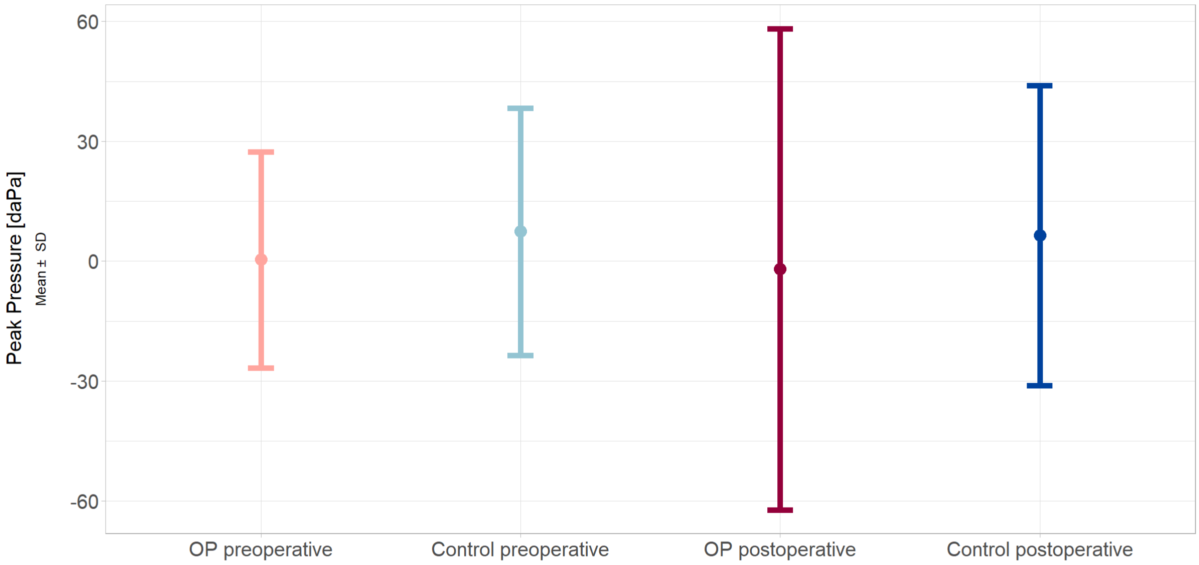

Figure S1. Graphical representation of pre- and postoperative peak pressures for the implanted (OP) and the control ear. Data is shown in *mean* for each measured frequency ± *SD*. The *mean* of the peak pressures was for all conditions around ambient pressure (OP preoperative: *Mean* = 0.39 daPa, *SD* = 27.0; Control preoperative: *Mean* = 7.42 daPa, *SD* = 30.93; OP postoperative: *Mean* = - 1.99 daPa, *SD* = 60.16; Control postoperative: *Mean* = 6.50 daPa, *SD* = 37.52). No difference was found between the peak pressures in the different groups (OP vs Control) at the two time points (pre- vs postoperative) (ANOVA: $F = 0.445$, $p = 0.72$)

Table S1. *Mean* and *SD* with results of the paired two tailed *t*- test for all statistically tested frequencies (0.5, 0.75, 1, 2, 3, and 4 kHz) for pre- and postoperative WBTA measurements in the OP and the control ear.

		**Control**				**OP**				**t- test**			
		Mean		*SD*		*Mean*		*SD*		*Est.*	*CI*	*t*	*p*
**500 Hz**	*Preop.*	0.39		0.12		0.36		0.14		- 0.26	- 0.09 0.04	- 0.84	0.41
	Postop.	0.40		0.12		0.35		0.08		0.01	- 0.04 0.06	0.41	0.69
	t-test	*Est.*	*CI*	*t*	*p*	*Est.*	*CI*	*t*	*p*				
		- 0.01	- 0.05 0.04	- 0.40	0.70	0.01	-0.04 0.06	0.41	0.67				
		Mean		*SD*		*Mean*		*SD*		*Est.*	*CI*	*t*	*p*
**750 Hz**	*Preop.*	0.66		0.14		0.59		0.19		-0.07	-0.15 0.01	-1.76	0.09
	Postop.	0.60		0.14		0.48		0.15		0.12	0.04 0.19	3.18	**< 0.01***
	t-test	*Est.*	*CI*	*t*	*p*	*Est.*	*CI*	*t*	*p*				
		0.06	-0.003 0.11	2.17	0.06	0.12	0.04 0.19	3.24	**< 0.01***				
		Mean		*SD*		*Mean*		*SD*		*Est.*	*CI*	*t*	*p*
**1000 Hz**	*Preop.*	0.76		0.16		0.70		0.17		-0.07	-0.14 0.002	- 1.98	0.06
	Postop.	0.70		0.16		0.60		0.18		0.10	0.03 0.17	2.75	**< 0.05***
	t-test	*Est.*	*CI*	*t*	*p*	*Est.*	*CI*	*t*	*p*				
		0.05	-0.006 0.10	2.32	0.05	0.10	0.04 0.17	3.10	**< 0.01***				
		Mean		*SD*		*Mean*		*SD*		*Est.*	*CI*	*t*	*p*
**2000 Hz**	*Preop.*	0.72		0.17		0.74		0.15		0.01	-0.05 0.07	0.34	0.73
	Postop.	0.73		0.17		0.76		0.20		-0.02	-0.11 0.07	-0.43	0.67
	t-test	*Est.*	*CI*	*t*	*p*	*Est.*	*CI*	*t*	*p*				
		-0.01	-0.09 0.07	-0.20	0.84	-0.02	-0.08 0.04	-0.80	0.43				

		Mean	*SD*			*Mean*	*SD*			*Est.*	*CI*	*t*	*p*
3000 Hz	*Preop.*	*0.73*	*0.19*			*0.71*	*0.24*			*0.02*	*-0.08 0.13*	*0.50*	*0.62*
	Postop.	*0.69*	*0.19*			*0.68*	*0.22*			*0.02*	*-0.08 0.12*	*0.36*	*0.72*
	t-test	*Est.*	*CI*	*t*	*p*	*Est.*	*CI*	*t*	*p*				
		0.05	*-0.04 0.14*	*1.26*	*0.22*	*0.03*	*0.03 0.17*	*2.99*	***< 0.01****				
		Mean	*SD*			*Mean*	*SD*			*Est.*	*CI*	*t*	*p*
4000 Hz	*Preop.*	*0.50*	*0.23*			*0.53*	*0.23*			*0.04*	*-0.04 0.12*	*1.05*	*0.30*
	Postop.	*0.46*	*0.23*			*0.42*	*0.20*			*0.03*	*-0.05 0.11*	*0.74*	*0.47*
	t-test	*Est.*	*CI*	*t*	*p*	*Est.*	*CI*	*t*	*p*				
		0.04	*-0.05 0.13*	*0.92*	*0.36*	*0.11*	*0.04 0.19*	*2.92*	***< 0.01****				
